# ‘You're Just Thinking About Going Home’: Exploring Person‐Centred Medication Communication With Older Patients at Hospital Discharge

**DOI:** 10.1111/hex.70065

**Published:** 2024-10-15

**Authors:** Henrik Cam, Kristin Franzon, Sofia Kälvemark Sporrong, Thomas Gerardus Hendrik Kempen, Cecilia Bernsten, Elisabet I. Nielsen, Lovisa Gustavsson, Elnaz Moosavi, Stina Lindmark, Ulf Ehlin, Maria Sjölander, Karl‐Johan Lindner, Ulrika Gillespie

**Affiliations:** ^1^ Department of Pharmacy Uppsala University Uppsala Sweden; ^2^ Department of Public Health and Caring Sciences Uppsala University Uppsala Sweden; ^3^ Utrecht Institute for Pharmaceutical Sciences Utrecht University Utrecht The Netherlands; ^4^ Geriatrics, Uppsala University Hospital Uppsala Sweden; ^5^ Östhammar Association of Relatives and Elderly People Östhammar Sweden; ^6^ Department of Pharmacy Region Västmanland Västerås Sweden

**Keywords:** aged, continuity of patient care, health communication, hospital discharge, observation, patient‐centred care, qualitative research

## Abstract

**Background:**

The hospital discharge process poses significant safety risks for older patients due to complexities in communication and coordination among stakeholders, leading to potential drug‐related problems post‐discharge. Adopting a person‐centred care (PCC) approach in medication communication by healthcare professionals (HCPs) is crucial to ensure positive health outcomes. This study aimed to explore the practice of PCC in medication communication between older patients and HCPs during the hospital discharge process.

**Methods:**

We conducted a qualitative study using non‐participatory direct observations of patient–HCP consultations during hospital discharge, followed by semi‐structured interviews with observed patients and, when applicable, their informal caregivers. Data collection occurred from October 2020 to May 2021 at two Swedish hospitals. We gathered data using an observational form and audio‐recorded all consultations and interviews. The data were analysed thematically using the systematic text condensation method.

**Results:**

Twenty patients were included (median age: 81 years [range: 65–94]; 9 female) in observations and 13 of them participated in interviews. Two patients were accompanied by an informal caregiver during the interviews. Three main themes were identified: (1) *The impact of traditional authoritarian structures*, depicts power dynamics between patients and their HCPs, showing how traditional structures influence the practice of PCC in medication communication during hospital discharge; (2) *Consultation timing and mode not on patients' terms*, describes suboptimal times and settings for consultations, along with the use of complex language that hinders effective communication; and (3) *Discrepancy in expectations of self‐care ability*, illustrates a mismatch between the self‐care guidance provided by HCPs during hospital discharge and the actual needs and preferences of patients and informal caregivers.

**Conclusion:**

Medication communication between older patients and HCPs during hospital discharge is frequently inconsistent with the practice of PCC. Not only must HCPs improve their communication strategies, but patients and their informal caregivers should also be better prepared for discharge communication and encouraged to participate in their care. This involvement would give them relevant knowledge and tailor communication to their individual needs, preventing problems in managing their medications after discharge.

**Patient or Public Contribution:**

An advisory group of six patients and/or informal caregiver contributors provided input on the study design, edited the consent forms, and helped develop the interview guide.

## 1 Background

The worldwide population is ageing and older persons face increased disease burdens, require more medications, and exhibit a higher likelihood of hospitalisation compared to middle‐aged adults [1]. Care transitions, which involve transferring care responsibilities for a patient from one healthcare provider to another, such as hospital discharge, are high‐risk situations for older patients [2]. Complexities in communication and coordination among stakeholders contribute to safety vulnerabilities [3, 4, 5], including the occurrence of drug‐related problems post‐discharge. Such problems can result in higher healthcare utilisation [6, 7] and economic burden [8]. Despite assumptions that patients can manage their medications and seek follow‐up post‐discharge, older patients are frequently insufficiently involved during hospital care, impairing their ability to resume responsibility [9, 10, 11, 12, 13, 14, 15, 16].

Effective communication between patients and healthcare professionals (HCPs) is vital for high‐quality healthcare and positive health outcomes [17, 18, 19]. Person‐centred care (PCC), which views patients as complete individuals rather than the traditional biomedical view of seeing them as diagnoses and passive recipients of treatment, is essential for enhancing communication [20, 21]. This approach involves placing the individual at the centre, considering their life, family, medical history, understanding of illness, needs, preferences, and experiences throughout their healthcare journey [20, 22]. While globally recognised as important [23, 24], implementing PCC in hospital settings is challenged by organisational factors, such as lack of support and leadership, as well as time constraints, and personal factors like cultural differences and inadequate knowledge [21, 25]. The complexity of managing multimorbidity in older patients further complicates the adoption of PCC when caring for this patient population [26, 27].

The *Medicines optimisation guidance* from the British Royal Pharmaceutical Society (2013) highlights the importance of a PCC approach for HCPs managing patients' medication therapy [28]. This approach was subsequently included in the NICE guideline for Medicines optimisation (2015) [29]. According to the guidance from the Royal Pharmaceutical Society, the overarching objective of PCC in relation to medication therapy is to empower patients to take ownership of their treatment. The theory outlines four guiding principles: (1) understanding the patient's experience, (2) evidence‐based medication selection, (3) maximising medication safety and (4) integrating medication optimisation into routine practice. By aligning with these principles, HCPs can facilitate improved outcomes, proper medication adherence, enhanced medication safety, and avoidance of unnecessary medications [28].

In Sweden, legislators have strived to adopt a PCC approach in legislation concerning medication optimisation in hospitalised patients [30]. However, it remains unclear how such an approach is intended to be implemented. Hospitals are required to provide the patient with an updated medication list and a discharge letter, which includes details of the hospitalisation course, the medication changes performed (including rationale for changes), and the follow‐up plans, all presented in layman's language and typically authored by the hospital physician [3, 30, 31]. A discharge summary containing similar information but intended for the next healthcare provider should also be written [3, 31]. In addition, it is customary for the hospital physician to conduct a dedicated oral discharge consultation with the patient before discharge. Despite these measures, gaps in PCC and patient involvement in medication decisions persist [10, 16, 32]. Therefore, there is a need to better comprehend the perspectives of older patients on PCC at hospital discharge, specifically regarding medication communication.

The aim of this study was to explore the practice of PCC in medication communication between older patients and HCPs during the hospital discharge process.

## 2 Methods

We employed a qualitative study design within a constructivist research paradigm, which claims that knowledge is constructed by individuals through their subjective interpretations of experiences and the context in which they occur [33]. To capture how communication is actually performed, we conducted non‐participatory direct observations [34] of patient consultations during hospital discharge, followed by semi‐structured interviews. These interviews, which concluded with a medication reconciliation, were conducted with the observed patients and, when applicable, their informal caregivers around one week after discharge. The medication reconciliation aimed to explore if the patients' actual medication use after discharge differed from what was prescribed. This study was part of a larger research project, Improved Medication Information and Patient Involvement at Care Transitions (IMPACT‐care), aimed at developing an intervention to enhance patient‐centeredness and medication safety at hospital discharge [35]. The Consolidated Criteria for Reporting Qualitative Research (COREQ) [36] were used in preparation of this manuscript.

### 2.1 Authors' Preunderstanding

The authors focused on PCC theory [20] and its application to medication therapy [28] throughout this study. These theories guided both the study design and the data analysis, ensuring a thorough exploration of patient perspectives concerning medication communication at discharge.

Most of the authors, except for two, have healthcare backgrounds, with several actively practising as clinical pharmacists, nurses, or physicians during the study. The progress of the study prompted these authors to reflect on their clinical roles in relation to patient communication. Of the two authors without healthcare backgrounds, one is a social scientist and the other has experience as an informal caregiver. Additionally, one author is a retired pharmacist and a public representative with political duties advocating for patients. The diverse backgrounds of the authors enriched discussions throughout the study. By involving the two authors who represented the public and informal caregivers, the research group maintained a strong focus on the patient perspective throughout the study.

### 2.2 Setting and Sampling

Patients were recruited from two publicly funded hospitals in different Swedish regions, Hospital A and Hospital B. Observations at Hospital A occurred at a geriatric and an internal medicine ward from October to November 2020, while those at Hospital B occurred at a cardiology ward for heterogeneity and pragmatic reasons from April to May 2021. A common electronic health record (EHR) system is shared between hospitals and primary healthcare centres within the two regions where this study was conducted. Consequently, hospital HCPs can access health records from primary care HCPs and vice versa.

Patients were included using a purposeful sampling method with a maximum variation strategy [33] based on patient gender, age, health complexity (assessed by the number of medications, changes, and diagnoses), and medication management support (self‐managed, supported by an informal caregiver, or using automated dose‐dispensing). Eligible patients were aged ≥ 65 years, home‐dwelling, managed their medications independently or with informal caregiver support before admission, and experienced at least one lasting medication change during hospitalisation. Patients with major neurocognitive disorders, in terminal care, or discharged to nursing homes were not eligible. Eligible patients were asked for participation (to be observed) by one of the researchers when their planned discharge date approached. Both patients and HCPs provided written informed consent before the observations, and patients and informal caregivers gave additional informed consent for the interview. The concept of information power [37] informed our sample size decision, with patient inclusion continuing until we determined that sufficient data had been collected from this perspective.

### 2.3 Data Collection

Observations were conducted by three of the authors: L. G. and E. M. (both female, final‐year pharmacy students), and M. S. (female, clinical pharmacist), supervised by H. C. (male, clinical pharmacist, trained in qualitative research) and U. G. (female, clinical pharmacist, trained in qualitative research). Before data collection, two pilot observations were carried out to standardise the observation process. During these pilots, the three observers (L. G., E. M., and M. S.) conducted the observations alongside H. C., K. F. (female, geriatrician), and U. G., and all discussed any differences in observations afterwards.

Patient consultations around planned discharge dates from 8.30 to 16.00 on weekdays involving physicians, nurses, and clinical pharmacists were observed. A patient consultation was defined as any interaction between an HCP and a patient. The observers waited outside of the patient's room to identify when a consultation was going to occur. They disclosed their background and wore uniforms identical to HCPs but with a prominent “observer” sign on their chest. Each patient was primarily observed by one observer during their hospitalisation. Observations were non‐participatory, meaning observers were present during the consultations but did not actively engage with the participants [34]. The observations, which focused on communication content, patient participation, and non‐verbal cues, were both audio‐recorded and documented using a previously developed data collection form by Rognan et al. [9], which included taking field notes. The form was translated to Swedish and minor contextual modifications were made to it (Supporting Information S1: File 1). The form consisted of a brief section of check‐boxes for gathering contextual data of the observation, with the majority of it comprising sections for note‐taking.

All observed patients, along with their informal caregiver if involved in medication management, were invited to participate in semi‐structured interviews about a week post‐discharge, conducted by H. C., U. G. or K. F. Originally planned as face‐to‐face sessions, these were switched to telephone interviews due to the Covid‐19 pandemic. An interview guide with open‐ended and prompt questions was developed by the researchers and public representatives based on the principles of PCC and our professional experience to facilitate the interview process (Supporting Information S2: File 2). The guide was piloted by H.C. through an interview with one discharged patient, leading to minor amendments to the questions. The pilot interview was not included in the study population. Interviews focused on patients' experiences with medication communication at hospital discharge and were audio‐recorded. The researchers reviewed notes from the observed consultations and written discharge documentation in the EHR before the interviews. At the end of the interviews, a medication reconciliation was performed to obtain structured information about the medications the patient was actually taking after returning home.

### 2.4 Data Analysis

The audio recordings were transcribed verbatim, and the field notes were digitally typed out. Data sources for each participant, including field note transcripts from the data collection forms, transcripts from observations and interviews, and medication discrepancies from medication reconciliations, were used for analysis. Thematic analysis was conducted following the systematic text condensation method [38], with the assistance of the NVivo (R1/2020) software. An inductive approach was employed, with regular consensus meetings held throughout the process. This analysis included the following steps:

1. H. C., U. G., and K. F. reviewed all the data from patients included at Hospital A.
2. For two patients with both observations and interviews, H. C., U. G., K. F. and S. K. S. (female, social scientist with an extensive background in health‐related qualitative research) individually coded the transcripts, followed by a consensus meeting to establish an initial coding scheme.
3. H. C., U. G. and K. F. coded all data from Hospital A, refining the initial coding scheme through consensus meetings as new codes, sub‐themes and main themes were identified.
4. An audit of the coding was conducted by S. K. S. and C. B. (female, public representative, retired pharmacist with an extensive background in health‐related qualitative research). Coded transcripts from three patients who underwent both observations and interviews were selected and reviewed to ensure that no relevant content was missed and that the coding was logical. Subsequently, a consensus meeting was held among H. C., U. G., K. F., S. K. S., and C. B. to make adjustments to the coding.
5. HC then coded all transcripts from patients at Hospital B, made adjustments as needed, condensed the coded content, and refined the main and sub‐themes.
6. A final consensus meeting attended by H. C., K. F., S. K. S., and T. G. H. K. (male, clinical pharmacist trained in qualitative research) was held to refine and confirm the main and sub‐themes. Quotes and patient examples (Table 3) were chosen to illustrate the themes.

### 2.5 Ethical Considerations

The study was approved by the Swedish Ethical Review Authority (Reg.no.: 2020‐02734). If any ethical issues arose during the study, such as while observing consultations or during medication reconciliation in patient interviews, the observer or interviewer was instructed to assist, provide advice, or contact the patient's healthcare provider. Although no serious issues emerged during the study, pharmaceutical advice was provided after the interviews to correct any misunderstandings. Data was pseudonymised and securely stored. All patient names in this paper are fictional.

## 3 Results

Twenty patients were asked and agreed to participate in observations: 14 from Hospital A and 6 from Hospital B (Table 1). Observational data comprised 42 patient consultations: 24 with physicians, 14 with nurses, and 4 with clinical pharmacists (Table 2). Physician consultations mainly consisted of discharge consultations, nurse consultations involved discharge coordination and medication dispensing, and pharmacist consultations focused on providing medication information. The overall median duration for a consultation was 11 min (range: 2–30 min), with a median duration of the physician's discharge consultation of 12 min (range: 4–30 min). Thirteen patients participated in post‐discharge interviews, lasting a median of 28 min (range: 12–53 min). Two patients were accompanied by an informal caregiver during the interviews. Those patients who did not participate were either too tired after returning home, unreachable, or rehospitalised. The physician's discharge consultation was observed for nearly all patients (18 out of 20), and 11 of these patients also participated in the interviews.

**Table 1:** Patient demographics.

Demographics	Hospital A (*n* = 14)	Hospital B (*n* = 6)	Total (*n* = 20)
Age, median years (range)	85 (66–94)	78 (65–80)	81 (65–94)
Sex			
Female	7	2	9
Male	7	4	11
No. of diagnoses in medical history, median (range)	5 (4–10)	3 (0–8)	5 (0–10)
Medication management before hospitalisation			
Automated dose‐dispensing at admission	3	0	3
Self	12	5	17
Informal caregiver support	2	1	3
Medications			
No. of medications at discharge, median (range)	14 (4–36)	9 (4–13)	13 (4–36)
No. of lasting^a^ medication changes at discharge, median (range)	6 (2–14)	4 (2–8)	6 (2–14)
Hospital environment			
Single‐bed room	3	6	9
Multibed room (sharing with 1 or 2 other patients)	11	0	11

^a^ Medication changes made during hospitalisation that lasted after hospital discharge.

**Table 2:** Summary of data collected for each included patient, indicating observed consultations at hospital discharge and conducted interviews after discharge.

Patient	Nurse consultation	Pharmacist consultation	Physician discharge consultation	Physician consultation, other	Interview
Adam (♂), 82 years	2; (9, 2)		1; (13)		1; (12)
Alex (♂), 83 years	1; (11)		1; (16)		
Anna (♀), 86 years			1; (23)		
Charlotte (♀), 90 years	1; (19)		1; (15)	1; (13)	1; (21)
Elias (♂), 65 years			1; (4)		1; (51)
Emma (♀), 85 years	2; (5, 4)	2; (5, 15)	1; (27)		
Eva (♀), 88 years		1; (14)	1; (14)		1; (18)
Felix (♂), 85 years	1; (11)				1; (28)
Gabriel (♂), 78 years	2; (6, 2)		1; (12)		1; (28)
Hanna (♀), 68 years			1; (13)		1; (43)
Helena (♀), 80 years			1; (5)	1; (7)	
Leo (♂), 66 years			1; (10)		
Liam (♂), 78 years			1; (10)		1; (33)^a^
Maria (♀), 84 years			1; (17)		1; (53)^a^
Olivia (♀), 75 years			1; (30)	1; (17)	
Oscar (♂), 80 years			1; (11)		1; (49)
Sarah (♀), 76 years			1; (9)		
Simon (♂), 95 years			1; (10)		1; (27)
Tomas (♂), 90 years	2; (2, 6)	1; (12)	1; (10)	1; (8)	1; (38)
William (♂), 76 years	3; (6, 12, 13)			2; (10, 6)	1; (17)
**Total *n* (median duration [range])**	14 (6 [2–19])	4 (13 [5–15])	18 (12 [4–30])	6 (9 [6–17])	13 (28 [12–53])

*Note:* The number of consultations is presented first in each cell, followed by the duration (in minutes) after the semicolon for each consultation or interview, provided in brackets.

^a^ An informal caregiver participated in the interview alongside the patient.

### 3.1 Main Themes and Sub‐Themes

**Figure 1:**
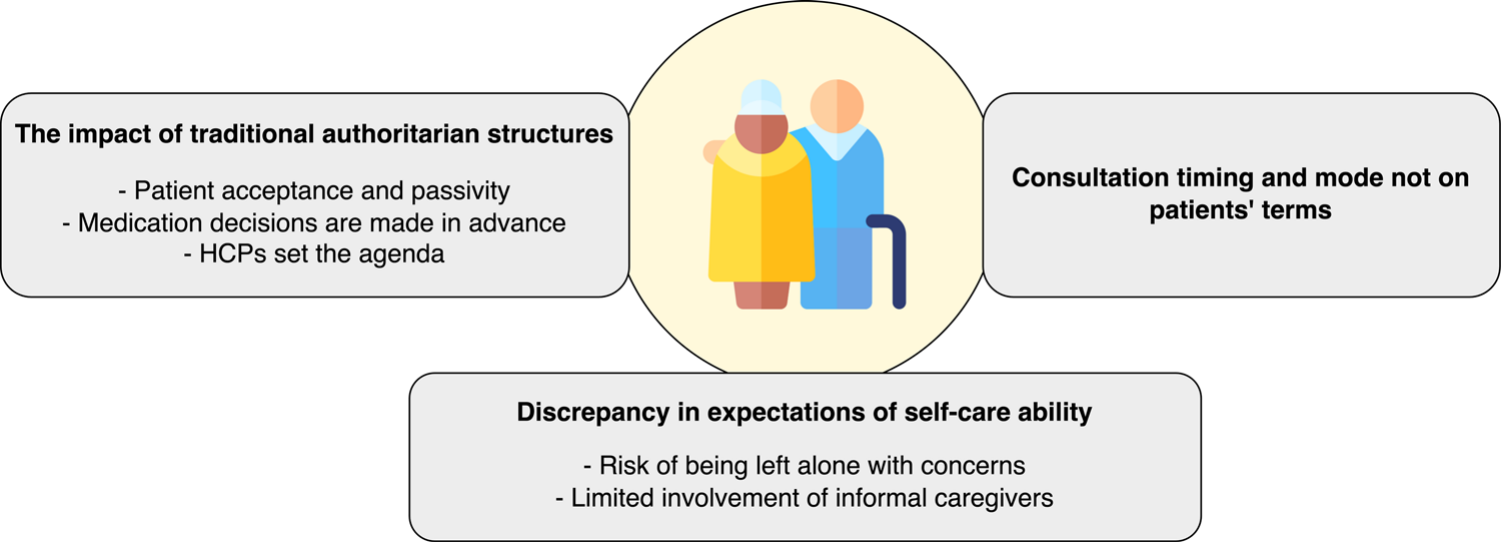
The three main themes, along with their sub‐themes describing the PCC in medication communication between patients/informal caregivers and HCPs during the hospital discharge process. HCP = healthcare professional, PCC = person‐centred care.

The analysis identified three main themes and several sub‐themes: (1) The impact of traditional authoritarian structures, (2) Consultation timing and mode not on patients' terms, and (3) Discrepancy in expectations of self‐care ability (Figure 1).

### 3.2 The Impact of Traditional Authoritarian Structures

Power dynamics between patients and their HCPs, shaped by the biomedical view and hence traditional authoritarian structures, influenced the implementation of PCC in medication communication during hospital discharge from both patient and HCP perspectives. This main theme included sub‐themes: (1) Patient acceptance and passivity, (2) Medication decisions are made in advance and (3) HCPs set the agenda.

#### 3.2.1 Patient Acceptance and Passivity

Many patients were satisfied with their hospital care and treatment, expressing high trust in HCP decisions and contentedly allowing HCPs to make decisions, believing these decisions to be appropriate. While they accepted prescribed medications and adapted to the care provided, some stressed the importance of receiving information at some point in the process. Some patients preferred being presented with medication choices based on expected effectiveness. However, due to a lack of knowledge, they felt unable to participate in medication decisions, finding it unimaginable to have a say.

I didn't really have a say; it [the medication decisions] just happened automatically/…/No, I'm an amateur so I have no idea, that's for the experts to handle, but a consultation is good.

Tomas, interview

Patients said that their queries were often addressed by HCPs during hospitalisation, yet many rarely asked questions during consultations. The consultations often concluded with the HCPs asking the patients if they had any questions, to which patients typically declined. Post‐discharge, however, several patients reflected on questions they wished they had asked, realising these concerns only afterwards. They sometimes desired different medications or increased dosages but felt unable to think of these questions during the discharge consultations because of uncertainties about how the medications would affect them after they returned home.

He [the physician] went through all the papers/…/point by point, I thought it [the discharge consultation] was very good, but there were some questions I might have wanted to ask, but I didn't really know how the medication would affect me, so I couldn't ask because I didn't know what to ask about.

Maria, interview

Despite the general trust in the HCPs among participants, doubts were occasionally expressed. One patient, for example, requested the reason for each new medication, eventually accepting them after a prolonged consultation with the physician.

I questioned why I should have that tablet/…/I was told that these two together have a much better effect/…/((sigh)) I just had to accept it but I don't like taking tablets.

Elias, interview

There were occasions where the authority of HCPs was evident, potentially reinforcing patient passivity. One example occurred during a consultation when the physician lifted the patient's shirt to check an ascites drainage bag, instead of asking the patient to do so or seeking permission beforehand, illustrating a perspective that viewed the patient more as a diagnosis than as a person.

The physician asks, ‘Do you still have that?’ and points to the patient's ascites drainage bag. The physician lifts the patient's shirt to examine the abdomen./…/says, ‘It looks fine’ and stops examining the patient, then stands with arms crossed/…/The patient still has the shirt pulled up, and the physician says, ‘This is from when you drank a lot of alcohol’. The patient looks away and says, ‘Yes’./…/[forward to the end of the consultation] The patient is still lying down with the shirt pulled up over the abdomen. The physician leaves the room, saying he will return.

Leo, observation of physician's discharge consultation

#### 3.2.2 Medication Decisions Are Made in Advance

Medication decisions were typically made by HCPs before consulting with the patient. During the consultations, there was an emphasis on informing the patients and motivating them to comply with the decisions already made. While HCPs often informed patients about medication changes, the explanations provided were typically brief. Changes were often grouped together and accompanied by a short description of the reasons for change. Information about the changes and related follow‐up plans appeared often to be communicated to patients for the first time upon discharge.

Physician: ‘Regarding the bone density treatment, we've discontinued the weekly tablet; instead, you'll receive an injection twice a year. The tablet can be quite large and impractical’.

Anna: ‘Well, it wasn't difficult for me to take/…/I know a relative who had that injection. But she's been given tablets instead now, I don't know why’

Physician: ‘It's hard to say/…/Then we've made another change, to your blood pressure treatment’.

Anna, observation of physician's discharge consultation

When patients inquired about the reasons for the changes, HCPs attempted to provide answers. However, if the answers were unavailable, they sometimes told the patients that another HCP with more expertise in the matter had made the decision previously, implying that further inquiries should be directed to them. However, they did not offer opportunities to discuss the matter with the decision‐maker. Following such exchanges, patients did not pursue the matter further.

Physician: ‘So the cardiologist has assessed that we'll discontinue it [rivaroxaban]/…/because the risk of bleeding for you is greater than the risk of getting a clot’.

Maria: ‘I understand/…/but I was so scared before since my mom and all her siblings died from heart attacks. When I came in [to the GP], he said, “You'll get this rivaroxaban so you won't get clots”’.

Physician: ‘Exactly, but rivaroxaban prevents clots in other places than in the coronary arteries/…/anyway, no blood thinners for now’.

Maria, observation of physician's discharge consultation

In a few cases, the HCP allowed the patient to determine their medication needs. Typically, these decisions were related to symptoms that the patients could easily recognise as worsening, such as pain or constipation. These patients responded positively about this experience during interviews, expressing relief in avoiding unnecessary medications.

Physician: ‘I'm thinking that you should continue with this newly prescribed tablet, oxycodone/…/or do you feel too dizzy or affected now, so that you'd prefer to stop taking it?’

Emma: ‘No, I don't want to stop, I'd like to try it at home first and see how it goes’.

Physician: ‘I think that's a wise decision’.

Emma, observation of physician's discharge consultation

#### 3.2.3 HCPs Set the Agenda

There was always a predefined agenda for the consultations, set by the HCP. While some consultations began with inquiries about the patient's well‐being and symptom improvements (patient case example Olivia in Table 3), occasions when the HCP addressed the patient's concerns were limited. The physician's discharge consultations typically began with the physicians announcing their intention to conduct a discharge consultation or to discuss the medication changes and events during the hospitalisation. The discharge summary often served as a reference, with the HCP guiding the patient through the written summary while discussing its contents. These consultations primarily focused on reviewing the care provided during hospitalisation, discussing medication changes made, and incorporating plans for follow‐up, either intermittently when changes were mentioned or at the end of the consultation. Generally, the medication discussions centred around the effects of medications rather than potential side effects. However, when side effects were mentioned, they were typically accompanied by direct advice or solutions to minimise their impact.

**Table 3:** Examples of patient cases where deficient or proficient person‐centred communication can contribute to risky or favourable scenarios.

Deficient person‐centredness contributing to risky scenarios		
*Patient background*	*Deficient person‐centredness*	*Risky scenario*
*William* ♂, *76 years* Medical history: Hypertension, heart failure, previously overweight Discharge diagnosis: Newly diagnosed type 2 diabetes Relevant medication changes: ○ Prescribed long‐acting insulin 24 IE once every evening ○ Prescribed metformin 500 mg twice daily ○ Prescribed sitagliptin 100 mg once daily Follow‐up planning: ○ Evaluation of the diabetes treatment at the GP	William was newly prescribed a long‐acting insulin during hospitalisation, and the HCPs planned for his discharge in the afternoon of the same day. However, during a consultation with the physician in the morning, it became apparent that he lacked knowledge on how to manage the insulin and measure his blood glucose levels. Throughout the day, a ward nurse, a specialised diabetes nurse and the physician provided William with information about the diabetes management on separate occasions. He practised how to inject a short‐acting insulin (which was discontinued before discharge) and measured his blood glucose levels once, but he still felt uncertain about managing his condition. Recognising William's uncertainty, the physician decided to postpone his discharge to allow more time to practise. However, he was eventually discharged the following day.	During the interview, William revealed that he had yet not administered the long‐acting insulin because he was unsure how to set the correct dose on the device. He also mentioned that he had not previously met the physician who discharged him over the weekend. He stated that he did not receive any discharge letter and the physician's discharge consultation felt rushed to him. As a result, William could not recall much from the consultation.
*Tomas* ♂, *90 years* Medical history: Heart failure, CKD, suspected COPD Discharge diagnosis: Newly diagnosed atrial fibrillation Relevant medication changes: ○ Prescribed apixaban 2.5 mg twice daily ○ Increased metoprolol dose from 25 to 50 mg once daily ○ Decreased allopurinol dose from 200 to 100 mg once daily ○ Discontinued furosemide 40 mg Follow‐up planning: ○ COPD evaluation at the hospital's pulmonary clinic ○ CKD evaluation at the hospital's nephrology clinic ○ Apixaban and other medication changes to be managed by the GP	On the morning of discharge, a clinical pharmacist provided Tomas with information about the medication changes, with a focus on the newly prescribed apixaban. At this time, he expressed a request for the follow‐up process to be simplified, as he found it too complicated to manage follow‐up appointments both at the hospital and with his GP. The pharmacist told Tomas that it may be necessary but advised him to discuss it with the physician later. Tomas restated this request to the nurse and during the discharge consultation with the physician. Despite his concerns, the physician explained to him that the planned follow‐up at different locations was necessary to ensure that the physician felt safe to discharge him.	During the interview, Tomas could only remember that he was newly prescribed apixaban; he did not recognise the other medication changes or understand the reason behind them. He still struggled to grasp the necessity of the complex follow‐up plans and expressed a desire to be followed up at a single location.

Proficient person‐centredness contributing to favourable scenarios		
Patient background	Proficient person‐centredness	Favourable scenario
*Hanna* ♀, *68 years* Medical history: Hypertension, spinal disc herniation Discharge diagnosis: Underwent heart surgery, received a biologic aortic valve prosthesis due to aortic valve disease and severe aortic stenosis Relevant medication changes: ○ Prescribed warfarin 2.5 mg once daily ○ Prescribed atorvastatin 40 mg once daily, replacing simvastatin ○ Prescribed furosemide 40 mg as needed ○ Prescribed oxycodone 5 mg as needed	The physician informed Hanna about warfarin during the discharge consultation. When discussing the follow‐up plan, she expressed concern about the difficulty of returning to the hospital clinic for warfarin monitoring due to the distance. The physician attentively listened to Hanna's concerns and promptly arranged for the monitoring to take place at her GP instead. Additionally, the physician provided her with the phone number to the hospital ward for any queries that may arise.	Hanna expressed during the interview that she was satisfied with the follow‐up planning and could clearly state the plans.
*Olivia* ♀, *75 years* Medical history: Hypertension, pulmonary embolism, psoriatic arthritis Discharge diagnosis: Pyelonephritis Relevant medication changes: ○ Prescribed felodipine 5 mg once daily ○ Prescribed spironolactone 25 mg once daily ○ Prescribed oxycodone/naloxone 10 mg/5 mg twice daily, replacing oxycodone 10 mg ○ Discontinued hydrochlorothiazide 12.5 mg once daily	It was the first time Olivia met the physician who conducted the discharge consultation. At the beginning of the consultation, before discussing the medication changes, the physician asked Olivia if there was anything on her mind and how she felt about returning home. She expressed her concerns to the physician, mentioning that she was slightly worried about returning home after being under hospital care for 2 weeks. The physician acknowledged Olivia's worries, reassured her that she had recovered well, and reminded her that the healthcare team, in collaboration with her, had decided that she was well enough to be discharged.	The duration of the initial conversation lasted around 2 min, after which the physician began informing about the medication changes. Olivia seemed focused on the consultation itself, as she actively participated by frequently asking questions and seeking clarifications on words she did not understand.

Abbreviations: CKD, chronic kidney disease; COPD, chronic obstructive pulmonary disease; GP, general practitioner; HCPs, healthcare professionals.

The overall consultation atmosphere appeared open, for example, allowing patients to interrupt and seek clarifications. However, some participants perceived the physicians' consultations as rushed, expressing concern over the limited time available. They highly valued interactions with other HCPs who had time to address their questions during hospitalisation. Conversely, others felt that they could not obtain answers from other HCPs as they often referred to the physicians. These participants appreciated the time that the physician took for the discharge consultation. It was observed that if patients deviated too much from the HCP's intended topic, their queries or concerns typically remained unanswered as the HCP redirected the conversation back to their original agenda.

#### 3.2.4 Consultation Timing and Mode Not on Patients' Terms

We noticed that the consultations often took place at suboptimal times and locations, and sometimes non‐layman language was used by HCPs, which is not coherent with effective communication, particularly for older patients.

At Hospital A, most consultations occurred in rooms with multiple patient beds, leading to frequent interruptions and disruptive noises from neighbouring patients, for example, nearby patients calling for HCPs' attention or other HCPs concurrently consulting them. Such interruptions were absent at Hospital B, where all patients had single‐bed rooms. Additionally, there were frequent instances of other HCPs interrupting ongoing consultations with unrelated tasks, such as blood sampling, dispensing discharge medications, or delivering meals.

The consultations typically took place with patients in their beds while the HCPs stood or sat nearby. Because consultations were conducted at patients' bedsides, they could easily be overheard by neighbouring patients in rooms with multiple patient beds. At times, HCPs initiated consultations while the patient was engaged in other activities, such as eating or sleeping. One common feature was that consultations often occurred abruptly for patients, based on the convenience of the HCPs.

Three physicians enter the patient's room and notice that the patient is not there. They knock on the bathroom door and ask if the patient is inside. The patient opens the door, and the physicians introduce themselves. They move to the bedside and wait. The patient exits the bathroom slowly/…/leaning on his crutch, and sits down./…/One of the physicians asks ‘You had COPD, right?’ The patient looks puzzled and replies ‘I've never heard that before’.

Tomas, observation of clinical rounds

There were also a few instances where the discharge consultation took place just outside the patient's bathroom. The physician quickly handed over the discharge documents, reviewed the written information, and left. Some patients pointed out that their hospitalisations, particularly the day of discharge, were hectic, leaving them mentally unprepared for comprehensive consultations with HCPs. They wished for consultations to be conducted differently than at bedside on the day of discharge to allow for better mental preparation.

I don't quite remember, but it was about the new medications, like Warfarin and stuff. There was so much [information]/…/when you've been lying there for 7–8 days, you're just thinking about going home/…./It is a bit confusing [when you are hospitalised] because they [HCPs] start early in the morning to take blood samples and there are things happening all the time.

Hanna, interview

Non‐layman language was often used by HCPs during patient consultations and we observed that patients mostly refrained from seeking clarification when faced with such terminology. While some HCPs recognised the potential challenge of the language and offered a clarification, others continued the conversation without further explanation. This lack of clarity may have contributed to post‐discharge queries, as some patients revealed during the interviews that they had not understood the information provided by HCPs during the consultations.

HCPs often communicated about medications implicitly, assuming that patients had prior knowledge. For instance, linking a substance to a diagnosis and planning a follow‐up without explicitly stating why it is needed presupposes that patients possess pre‐existing knowledge about the effects of a medication, which may not always be the case.

Physician: ‘We increased your bisoprolol dosage, which you take for atrial fibrillation, because your heart rate was a bit fast/…/I've asked your GP to make a follow‐up, to check that your heart beats well and all that’.

Adam, observation of physician's discharge consultation

### 3.3 Discrepancy in Expectations of Self‐Care Ability

There was a difference between the self‐care instructions that HCPs provided for patients and informal caregivers to smoothly resume self‐care, and what patients and informal caregivers actually needed. This indicates misalignments between the guidance given by HCPs and the practical needs and preferences of patients and caregivers. This main theme encompasses the following two sub‐themes: (1) Risk of being left alone with concerns and (2) Limited involvement of informal caregivers.

#### 3.3.1 Risk of Being Left Alone With Concerns

Most patients found the consultations with HCPs before discharge valuable for reassurance and discussion, allowing them to have their questions answered. However, during the interviews, patients varied in their recall of the information from the consultations. While a few could effortlessly recall medication changes and follow‐up plans from the discharge summary, others struggled to remember the details. Some patients, although finding the consultations clear, felt overwhelmed by the amount of information and forgot it once they returned home. Others could not even recall having consultations or receiving written discharge documents. Those who remembered receiving discharge documents found them beneficial for their comprehensive information and the opportunity to review it at home.

Patients often received comprehensive medication instructions during consultations, where the importance of obtaining necessary medications to avoid treatment gaps after discharge was emphasised. Some patients, despite receiving the same information repeatedly from different HCPs, still struggled to grasp it (patient case examples William and Tomas in Table 3).

Patients were routinely informed about follow‐up plans predetermined by the healthcare team, particularly during the physicians' discharge consultations. These plans typically included where and when follow‐up should occur, although the specific timing was often vague. On some occasions, patients received certain follow‐up responsibilities by physicians, such as scheduling appointments for blood pressure measurement or adjusting medication doses. A few patients requested simplification of the follow‐up procedure after discharge, and HCPs accommodated these requests when deemed feasible from their perspective (patient case examples – Hanna and Tomas in Table 3). At times, patients expressed concerns about their medications or self‐care after discharge during consultations. Instead of further addressing and investigating these concerns, HCPs often relied on medical guidelines to justify their decisions and attempted to reassure patients by mentioning the accessibility of their EHR to subsequent healthcare providers and arrangements for follow‐up with the patient's GP would be made.

Emma: ‘I know that it [nitrazepam] helped so well/…/now I may have to keep trying something else that is worse, and that is not fun either ((sadness in her voice))’.

Physician: ‘Nitrazepam is a very strong medication that we prefer not to prescribe for older persons’

Emma: ‘But I've had it for almost thirty years, so I could just continue with that’.

Physician: ‘Well, I agree with you, but I still think we have other medications left to try first’.

Emma: ((heavy emphasised exhale))

Physician: ‘/…/but you will receive a document summarising everything and we'll inform your GP about how we're thinking’.

Emma, observation of physician's discharge consultation

During the interviews, some patients still had concerns about their medications and were awaiting the follow‐up appointment with their GP, typically assuming these were arranged by the hospital HCPs and trusting they would be notified when the time came. However, in the meantime, they mentioned handling these concerns differently. Some expressed frustration at the lack of clarity and resumed medication treatment as they thought was correct, while others mentioned seeking information online and felt confident in doing so. Knowing that a follow‐up was planned provided them with reassurance and a clear understanding of what to expect. However, they often remained uncertain about what the follow‐up entailed, including location, procedure, and timing, leaving them concerned.

Informal caregiver: ‘He's supposed to have a follow‐up at the outpatient medicine clinic, so he'll probably be called eventually, I hope’.

Interviewer: ‘Do you know what it is that they're supposed to follow‐up on?’

Informal caregiver: ‘No, it [the discharge letter] just says follow‐up/…/’

Liam: ‘No, I don't remember if the physician said anything about it’.

Liam and informal caregiver, interview

#### 3.3.2 Limited Involvement of Informal Caregivers

During interviews where informal caregivers participated, both patients and their informal caregivers emphasised the importance of the involvement of the informal caregiver for a seamless continuity of the medication treatment after discharge. Despite this, no informal caregivers participated in the observed consultations. Two of the informal caregivers mentioned receiving a phone call from a nurse before the patient's discharge, where they were informed about medication changes and given an opportunity to ask questions. One informal caregiver stated that there was no prior communication with HCPs before discharge. Instead, information was received through the discharge documents brought home by the patient. Upon reading the documents, the informal caregiver had questions that the patient could not answer and it was challenging to get answers from HCPs afterwards.

Informal caregiver: ‘They could have phoned and informed me about any specific concerns regarding the medications/…/he [Liam] could apparently take all the medications at once [in the morning] except for one tablet that he should take in the evening, but it would be good to receive a heads up so you don't give them all at once’.

Liam and informal caregiver, interview

The informal caregivers highlighted the crucial need of receiving information from the HCPs and stated that it was a necessity for them to accept the medication changes made to the patient's regimen. Although they did not consider themselves knowledgeable enough to participate in medication decisions, they expressed high engagement in the patients' care during hospitalisation and actively sought ways to stay informed.

Informal caregiver: ‘We haven't received that [the discharge letter] however, I can go online and look into her EHR and see what has happened./…/I have looked now at the end [of the hospitalisation] what they [HCPs] have written./…/I haven't really influenced the care, I don't have the expertise to do that regarding medications’.

Maria and informal caregiver, interview

## 4 Discussion

This qualitative study provides insights into the practice of PCC in medication communication between older patients and HCPs during the hospital discharge process. Three main themes were identified: *The impact of traditional authoritarian structures*, *Consultation timing and mode not on patients' terms*, and *Discrepancy in expectations of self‐care ability*. The findings reveal that although the medication communication at discharge often was comprehensive in terms of information content, it frequently failed to align with PCC practices. Communication seems to typically involve a one‐way transfer of information from HCPs to patients, with consultations conducted more according to the preferences of HCPs than those of the patients. Consequently, patients may face challenges in managing and adhering to their medications post‐discharge.

### 4.1 Challenges to Practise PCC in Medication Communication at Hospital Discharge

The main themes, *The impact of traditional authoritarian structures* and *Consultation timing and mode not on patients' terms*, highlight substantial power imbalances between older patients and HCPs, accompanied by suboptimal conditions, such as complex language, interruptions and disturbing noises. These factors adversely affect PCC in medication communication and illustrate the traditional biomedical model's dominance [27, 39].

A consistent finding across all three main themes is that HCPs primarily focused on providing information, whereas patients prioritised receiving it. In terms of engaging in medication decisions, patients and informal caregivers felt unable to participate due to limited knowledge. This disengagement presents a critical barrier to PCC in medication communication, as active patient participation in medical decisions is considered a prerequisite for PCC [22]. Older patients' unwillingness to participate actively in their treatment and their tendency to readily accept decisions made by HCPs have been reported in similar studies [32, 40]. Although active patient participation is a relatively recent expectation and one that patients may not yet be accustomed to [25], there are findings suggesting that patients' reluctance may also stem from not being invited to participate, rather than a lack of interest to engage in their care [25]. Our main themes, *The impact of traditional authoritarian structures* and *Discrepancy in expectations of self‐care ability*, support this viewpoint. They depict situations where patients' concerns were not adequately addressed by HCPs, who approached consultations with a biomedical focus on transmitting predetermined information from the discharge documents and failing to adapt to the patient's individual needs. This biomedical approach limited patients' opportunities to express themselves, further confirmed by the relatively short median duration of physician's discharge consultations (12 min) and high mean number of medication changes at discharge (6 changes) among the study population. The short duration in combination with multiple medication changes may have contributed to the rushed consultations, where there is little time for patients to voice their concerns effectively. These findings resonate with those of Rognan et al. [41], who observed that even patients actively seeking involvement in their care could not shift the fixed mindset of HCPs, resulting in persistently suboptimal communication. In contrast, situations where PCC‐influenced communication strategies are applied, such as initiating consultations by asking the patients about their concerns and information needs (see Table 3), lead to higher patient satisfaction, understanding, and health outcomes [18]. Therefore, these strategies are examples considered to be best practices for person‐centred communication [18].

### 4.2 Unprepared Patients and Communication Gaps

The fact that patients felt unprepared for consultations is reinforced by findings from the main themes, *Consultation timing and mode not on patients' terms* and *Discrepancy in expectations of self‐care ability*. Consultations occur so abruptly that patients are unprepared and left uncertain about what questions to ask or what information is crucial for continuing care after discharge. Consequently, many do not recall the details discussed during these consultations. Aware of the extensive information they must convey to patients, HCPs seem to place considerable reliance on the written discharge letters given to patients [42, 43]. However, these letters often contain deficiencies [44] and might not include information important to the individual patient, complicating patients' ability to manage their medication post‐discharge. This situation is exacerbated by patients and informal caregivers mistakenly assuming that follow‐up arrangements are automatically and always correctly handled by HCPs, leaving them uninformed about the specifics of these follow‐ups, as depicted in the main theme, *Discrepancy in expectations of self‐care ability*. This is concerning because the transfer of follow‐up requests to the next healthcare provider is also known to be deficient [45], posing a risk to effective treatment and patient safety. While informal caregivers could serve as crucial support for patients, this main theme describes that they are not adequately involved in the discharge care processes, a finding consistent with previous research indicating insufficient caregiver involvement at hospital discharge [13].

Common misconceptions about the PCC concept, such as the belief that patients should make all decisions about their healthcare or the assumption that patients prefer HCPs to make decisions, contribute to difficulties among HCPs to understand why and how PCC should be applied in medication communication [25]. However, when HCPs align with PCC principles, they often describe the experience as beneficial and rewarding, despite the challenges involved [46]. Another main challenge for HCPs in practising PCC in medication communication is the pressured hospital care environment, which often leads to hurried discharge processes that are not well‐adapted to individual needs [3]. In this high‐pressure setting, even when HCPs recognise the advantages of the PPC approach in patient communication, they may default to the biomedical model, as it is perceived to be less demanding [46]. Consequently, the focus tends to shift towards discharging patients quickly rather than adequately preparing them for self‐management at home [10, 15].

### 4.3 Future Clinical Strategies to Facilitate PCC by Increasing Patient Participation

Effective communication aligned with PCC practices is essential to empower patients to manage their own care after discharge [47]. The current lack of PCC during medication communication signals a need for adjustments, not only in how HCPs communicate but also in enhancing patients' abilities to actively participate in their care. Crucial to this is training HCPs in specific communication strategies, including actively listening to and addressing patient concerns, and minimising medical jargon [18]. Additionally, it is important to foster patient engagement and knowledge. Encouraging patients to participate actively in their care by asking questions, expressing preferences, and involving them in planning follow‐up care are fundamental components of PCC [20]. Tailoring the timing of discharge consultations to better meet the patients' needs, considering quieter, more comfortable settings like their home environment or a dedicated discharge consultation room could enhance the usefulness of the consultations. Supporting informal caregiver involvement in the discharge process is equally important. By providing informal caregivers with the same education and instructions as patients, caregivers can be better prepared to support the patient's post‐discharge care [48, 49]. Future studies should explore ways to incorporate these enhanced patient participation strategies in interventions to better prepare older patients for returning home.

### 4.4 Methodological Considerations

A key strength of our study is the combination of direct observations and subsequent semi‐structured patient interviews, allowing us to capture richer data than interviews alone would provide. Additionally, the diversity within our research group in terms of professional backgrounds, genders and ages, as well as the inclusion of public and informal caregiver representatives, further strengthens our study. The diversity enhanced the trustworthiness and credibility of our findings through investigator and analyst triangulation [33].

The observer effect, a known limitation of direct observational methods, could have influenced the behaviour of patients and HCPs [50]. To mitigate this, we employed students as observers. Svensberg et al. suggest that such observers do not considerably alter hospital HCPs' behaviour, as they are accustomed to students shadowing them [51]. Another limitation was that we only observed consultations around planned discharge dates from 8.30 to 16.00 on weekdays. Consequently, we cannot rule out that patients might have been invited to participate in medication decisions during consultations that were not observed, potentially reducing the necessity for HCPs to invite participation again during the observed consultations. However, neither observed patient behaviour suggested prior discussions with HCPs nor did patients indicate this during the interviews.

Purposeful sampling allowed the inclusion of patients with varied backgrounds in terms of gender and medical history, increasing the information power and reducing the risk of missing relevant aspects for our research aim.

Data collection occurred during the Covid‐19 pandemic, a period of significant healthcare strain and visitor restrictions, potentially impacting the transferability of our findings to other times. However, the observations were conducted during a relatively low‐intensity phase of the pandemic, and care was largely provided as usual. Substantial pressure on the healthcare system existed both before and after the pandemic, suggesting a consistent context. Transitioning from face‐to‐face to telephone interviews might have reduced interview depth [52], but the median duration (28 min) indicates that it did not substantially affect patients' ability to share their thoughts.

## 5 Conclusions

This qualitative study revealed that medication communication between older patients and HCPs during hospital discharge is frequently inconsistent with the practice of PCC. Not only must HCPs improve how they communicate but there is also a need for a broader change in how medication communication is approached. Patients should be proactively prepared for hospital discharge communication and encouraged to take an active role in their care. This involvement would allow them to gain knowledge and tailor communication to their individual needs, preventing problems in managing and adhering to medication regimens after discharge.

## Author Contributions

**Henrik Cam:** conceptualisation, formal analysis, project administration, writing–original draft, investigation, methodology, writing–review and editing. **Kristin Franzon:** conceptualisation, formal analysis, funding acquisition, project administration, investigation, methodology, writing–review and editing. **Sofia Kälvemark Sporrong:** conceptualisation, formal analysis, methodology, writing–review and editing. **Thomas Gerardus Hendrik Kempen:** conceptualisation, formal analysis, funding acquisition, methodology, writing–review and editing. **Cecilia Bernsten:** conceptualisation, methodology, funding acquisition, formal analysis, writing–review and editing. **Elisabet I. Nielsen:** conceptualisation, methodology, funding acquisition, writing–review and editing. **Lovisa Gustavsson:** conceptualisation, methodology, investigation, writing–review and editing. **Elnaz Moosavi:** conceptualisation, methodology, investigation, writing–review and editing. **Stina Lindmark:** conceptualisation, methodology, writing–review and editing. **Ulf Ehlin:** conceptualisation, methodology, writing–review and editing. **Maria Sjölander:** conceptualisation, methodology, investigation, writing–review and editing. **Karl‐Johan Lindner:** conceptualisation, methodology, funding acquisition, writing–review and editing. **Ulrika Gillespie:** conceptualisation, methodology, funding acquisition, investigation, formal analysis, project administration, writing–review and editing.

## Ethics Statement

The study was approved by the Swedish Ethical Review Authority (Reg.no.: 2020‐02734).

## Consent

Patients and HCPs provided written informed consent before the observations, and patients and informal caregivers gave additional informed consent for the interview.

## Conflicts of Interest

The authors declare no conflicts of interest.

## Supporting information

Supporting information.

Supporting information.

## Data Availability

Upon request and with approval from the IMPACT‐care research group, the corresponding author can provide access to the pseudonymised transcripts used for analysis in the present study.
