# Supplementary material for: ‘You're Just Thinking About Going Home’: Exploring Person‐Centred Medication Communication With Older Patients at Hospital Discharge
Source: Health Expect. 2024 Oct 15;27(5):e70065. doi: 10.1111/hex.70065 (PMC11474703; doi:10.1111/hex.70065)
Supplement: Supplementary file 1 — Supporting information. [file HEX-27-e70065-s002.pdf]

Supplementary file 1: Data collection form (translated to English)

Data collection form					Page
**Record ID:**	**Hospital:**	**Ward:**	**Room:**	**Observer:**	**Date:**

Current information from medical records (prescribed/discontinued medications, changed doses/formulations, and other details)

**Time start observation**	**Time finished observation**	**Audio recording** ☐
**Description of surroundings**<br>☐ Single bed<br>☐ Multiple bed<br>☐ Other<br>___________________________	**HCP observed**<br>Profession______________________<br>☐ **Consent**<br>☐ Male ☐ Female	**Consultation type**<br>☐ Discharge<br>☐ Other_______________________<br>☐ Samples/monitoring<br>☐ Patient called<br>☐ Medication dispensing

Observer's notes

Instructions:

1. Chronological observation of the content of the consultation and the interaction between patient and HCP (sequence of events, quotes, non-verbal cues); description of the environment/situation.
2. Interpretations of the observation; perception of patient-HCP interaction; patient involvement; any potential impact of the observer on the conversation.

Initial observation of patient and healthcare personnel's starting point:

Data collection form					Page
**Record ID:**	**Hospital:**	**Ward:**	**Room:**	**Observer:**	**Date:**

Observer's notes

Instructions:

1. Chronological observation of the content of the consultation and the interaction between patient and HCP (sequence of events, quotes, non-verbal cues); description of the environment/situation.
2. Interpretations of the observation; perception of patient-HCP interaction; patient involvement; any potential impact of the observer on the conversation.
