# Supplementary material for: ‘You're Just Thinking About Going Home’: Exploring Person‐Centred Medication Communication With Older Patients at Hospital Discharge
Source: Health Expect. 2024 Oct 15;27(5):e70065. doi: 10.1111/hex.70065 (PMC11474703; doi:10.1111/hex.70065)
Supplement: Supplementary file 2 — Supporting information. [file HEX-27-e70065-s001.docx]

**Supplementary file 2 (translated to English)**

**Interview guide for patients/informal caregivers**

**Information from the interviewer**

- Explain the study's purpose of exploring the patient's perspective on medication communication before leaving the hospital. Encourage the patient/family member to respond as openly and honestly as possible and to clarify any unclear questions.
- Inform about the consent granting access to medical records (handled confidentially).
- Inform that the interview is expected to take 30-60 minutes, will be recorded and pseudonymised for publication.
- Clarify the researcher's role: "I'm interviewing you in my role as a researcher, but I also work as a pharmacist/physician. So if I notice any misunderstandings about your medication treatment during the interview, we can discuss it afterward."

**Medication Communication at Discharge**

1. What information about your medication treatment did you receive before leaving the hospital?
2. How was the information about your medication treatment that you received before being discharged from the hospital?
3. Were there any changes in your medication treatment during your hospital stay?
   - Follow-up question: Do you know why these changes were made?
4. What information did you receive about how the changes in your medication treatment will be followed up?
   - Follow-up question: Was the information sufficient/insufficient? Was it easy/difficult to understand?
5. Have you been in contact with the primary care centre or hospital after being discharged from the hospital?
   - Follow-up question: Who do you contact if you have questions about your medication?

**Discharge consultation with the physician**

1. Think back to the consultation with the doctor before you went home. Can you tell me about it, what was discussed regarding medication?
   - Follow-up question:
     - Did the doctor go through which medications you should take, how often, and how much with you?

**Patient involvement**

1. How involved do you feel you have been in decisions about your medications?
2. How do you feel about deciding and more responsibility for your medication treatment?
3. How do you feel about taking more responsibility for the follow-up of your medication treatment?

**Improvement Potential**

1. What was good and bad about the information you received about medication at discharge?
2. What do you think could be improved regarding how you received information about your medication in the hospital?

**Medication Reconciliation**

1. What medications do you take and in what dosage?
2. How do you keep track of which medications you should take?

**Other**

1. Have you thought of anything you would like to add to what we discussed?

***Handle and correct any misunderstandings about medication treatment discovered during the interview.***
